# Novel and pragmatic exploration of variation in glottic parameters in non-parallel versus parallel vocal cord CT planes with potential reporting pitfalls

**DOI:** 10.1371/journal.pone.0293659

**Published:** 2023-10-30

**Authors:** Adeena Khan, Waleed M. S. Fawzy, Syed S. Habib, Mamoona Sultan

**Affiliations:** 1 Department of Radiology and Medical Imaging, King Saud University, Riyadh, Saudi Arabia; 2 Department of Physiology, King Saud University, Riyadh, Saudi Arabia; 3 Department of Emergency Medicine, King Saud University, Riyadh, Saudi Arabia; University Hospital Eriangen at Friedrich-Alexander-University Erlangen-Numberg, GERMANY

## Abstract

Oblique orientation of vocal cord demands strict compliance, by technicians and clinicians, to the recommended parallel plane CT scan of larynx. Repercussions of non-compliance has never been investigated before. We aimed to observe influence of non-parallel vocal cord plane CT scan on qualitative and quantitative glottic parameters, keeping parallel plane CT as a standard for comparison. Simultaneous identification of potential suboptimal imaging sequelae as a result of unformatted CT plane was also identified. In this study we included 95 normal adult glottides and retrospectively analyzed their anatomy in two axial planes, non-parallel plane ① and parallel to vocal cord plane ②. Qualitative (shape, structures at glottic level) and quantitative (anterior commissure ACom, vocal cord width VCw, anteroposterior AP, transverse Tr, cross-sectional area CSA) glottic variables were recorded. Multivariate statistical analysis was used to predict pattern and their impact on glottic anatomy. Plane ① displayed supraglottic features in glottis; adipose (90.5%) and split thyroid laminae (70.6%). Other categorical variables: atypical shape, submental structures and multilevel vertebral crossing were also in majority. All glottic dimensions varied significantly between two planes with most in ACom (-5.8mm) and CSA (-15.0 mm^2^). In contrast, plane ② manifested higher VCw (>73%), Tr (66.3%), CSA (64.2%) and AP (44.2%) measurements. On correlation analysis, variation in ACom, CSA, Tr was positively associated with VC or plane obliquity (p<0.05). This variability was more in obese and short necked subjects. Change in one parameter also modified other significantly i.e., ACom versus AP and CSA versus Tr. Results indicated statistically significant change in subjective and objective anatomical parameters of glottis on non-application of appropriate CT larynx protocol for image analysis hence highlighting importance of image reformation.

## Introduction

Selection of a pertinent tomographic plane according to region of examination, for synchronization of gross and radiological anatomy is a pre-requisite for cross-sectional imaging. Ignorance to the set CT protocol in other body sites may not be as consequential as in larynx [1–4]. This has been a matter of discussion since many decades and is more challenging in case of CT larynx owing to its complex anatomy, narrow interspace of transition between its three subdivisions (supraglottis, glottis and subglottis) and dynamic nature during different phases of breathing and phonation [5, 6].

Following laryngoscopic evaluation, CT scan is recognized as a prime ancillary investigation along with promising role of modified laryngeal techniques [7–10]. To bring pre-selected axial plane of scout CT scan in alignment with vocal folds, it is suggested to tilt either gantry or head position parallel to cord plane [11, 12]. Evolution of CT scan machines brought facility of multiplanar planar reformatting (MPR) which allows modification of scan plane in any orientation with achievement of higher quality diagnostic images [5].

Glottis has a primary role in larynx because of its pivotal contribution in voice production and supporting role in breathing [13]. Diagnostic accuracy of CT scan to gauge involvement of adipose tissue in laryngeal spaces, laryngeal cartilaginous framework and anterior commisure ranges between 75–88% [7, 14]. Multidirectional evaluation of normal airway lumen size on CT scan is a principal predictor for selection of endotracheal tube and management of stenosis [15, 16]. Correct anatomical description on imaging is fundamental to various cosmetic glottic procedures, tumor surgeries, airway management and radiotherapy planning [5, 16–20]. All these are sensitive to changes in CT plane. Out of three planes, axial planar reformat has shown to dispense substantial information about glottic structures [5, 21]. Erroneous and inaccurate representation of healthy glottis anatomy, localization of pathology and description of quantitative parameters in this plane can misdirect management decisions [9, 14]. It necessitates involvement of a radiologist to ensure that CT images are conforming to true vocal cord plane and hence representative of endoscopic and surgical anatomy for an optimal patient management [6, 9]. This can only be sustained if we have comprehensive knowledge about normal glottic anatomy along with broad concept of its subjective and objective anatomical alterations as a sequelae of non-compliance to an accurate CT protocol.

To best of our knowledge, we have not encountered any research addressing an importance of accurate tomographic plane in CT larynx. This article aims to report variations in different glottic anatomical parameters by comparing them in two axial planes i.e., non-parallel and parallel to vocal cord (NPVC/plane①, PVC/plane②). Moreover, quantitative description of glottis in true plane and assessment of potential pitfalls leading to misdiagnosis are also identified which might be used for future reference because laryngeal measurements taken on autopsy samples are not true exemplification of live laryngeal model due to limitation of tissue changes [22].

## Materials and methods

### Patient population

This retrospective study was approved by institutional review board of King Saud University. Retrospective data of all patients was completely anonymized and consent was not required. Institutional approval from Research Ethics Committee of King Saud university (Ref. No. 21/0428/IRB) was obtained. All contrast enhanced neck CT (CECT) scans available between year 2017–2021 were included in sample after surveillance for their inclusion and exclusion criteria (Table 1). Primarily, demographic data and body mass index (BMI) were taken from patient’s record followed by imaging analysis.

**Table 1:** Exclusion criteria for our patient selection.

Exclusion criteria
• ≤ 18 years • Missing data of patients • CT neck without contrast • Slice thickness >3mm. • Inadequate CT technique (adducted vocal cord, artifacts, suboptimal neck position) • Any laryngeal or cartilage pathology • Vocal cord paralysis • Post-surgical, post-traumatic larynx • Neck mass causing mass effect on larynx

### CT scan protocol

**Fig 1:**
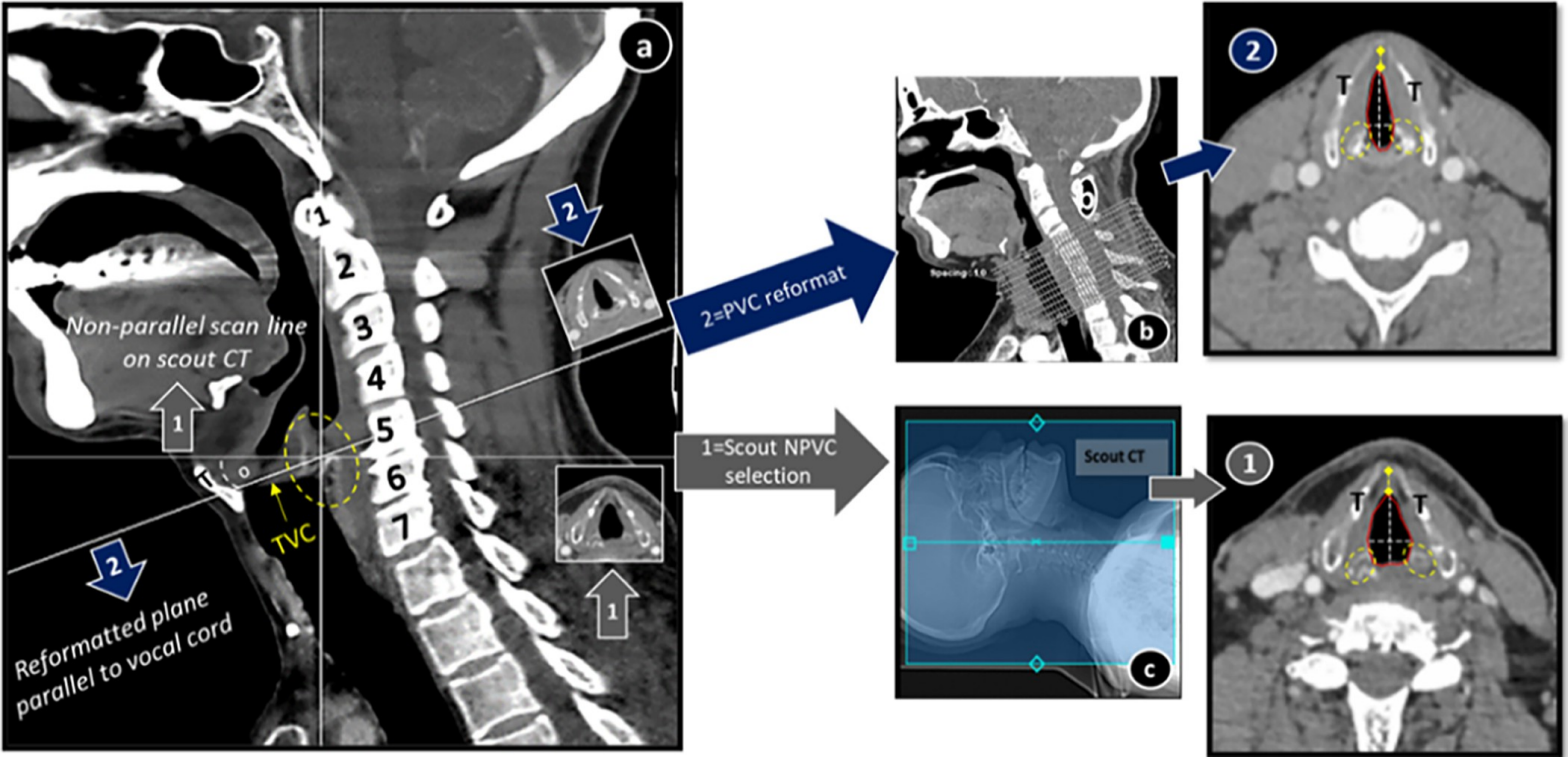
CT scan neck sagittal section (a) showing how glottic anatomy was studied in two axial planes, non-parallel, NPVC ①and parallel to vocal cord PVC ②. Scout CT (c) is showing predefined non-parallel CT scan lines and reformatted CT scan (b) showing PVC reformation lines in sagittal. Qualitative and quantitative parameters of glottis noted in both ① and ②. Dotted oval = cricoarytenoid joint (a,1,2), o = angulation between two planes (a), TVC = true vocal cord, red lumen trace = Cross-sectional area, dotted white lines = anteroposterior and transverse dimensions, double arrow line = anterior commisure, yellow dots = region of vocal cord width measurement.

GE (General Electric) CT scanner (GE revolution CT 256 multidetector) was utilized. Neck scanning protocol was supine position with neutral or slightly extended neck, as per patient’s compliance. Extent of coverage area was from base of skull to arch of aorta while patient was instructed to take quiet breathing and not to swallow during scan time. 60 mL intravenous contrast (omnipaque 300-lohexol) was administered at the rate of 3 milliliter/second and scanning initiated after 45 seconds delay. Scanning parameters included 120 kVp (kilovoltage peak), automated tube current, 0.5-second rotation time, FOV (field of view) 25 cm, pitch 0.8 and 512×512 pixel matrix. Initial scanning was done in plane perpendicular to CT table (Fig 1C①). Images raw data was then transferred to GE workstation for MPR by using 0.625 mm helical images with 1–3 mm reconstruction in plane parallel to vocal cord without re-exposure to radiation (Fig 1B②).

### CT imaging analysis (Figs 1 and 2)

**Fig 2:**
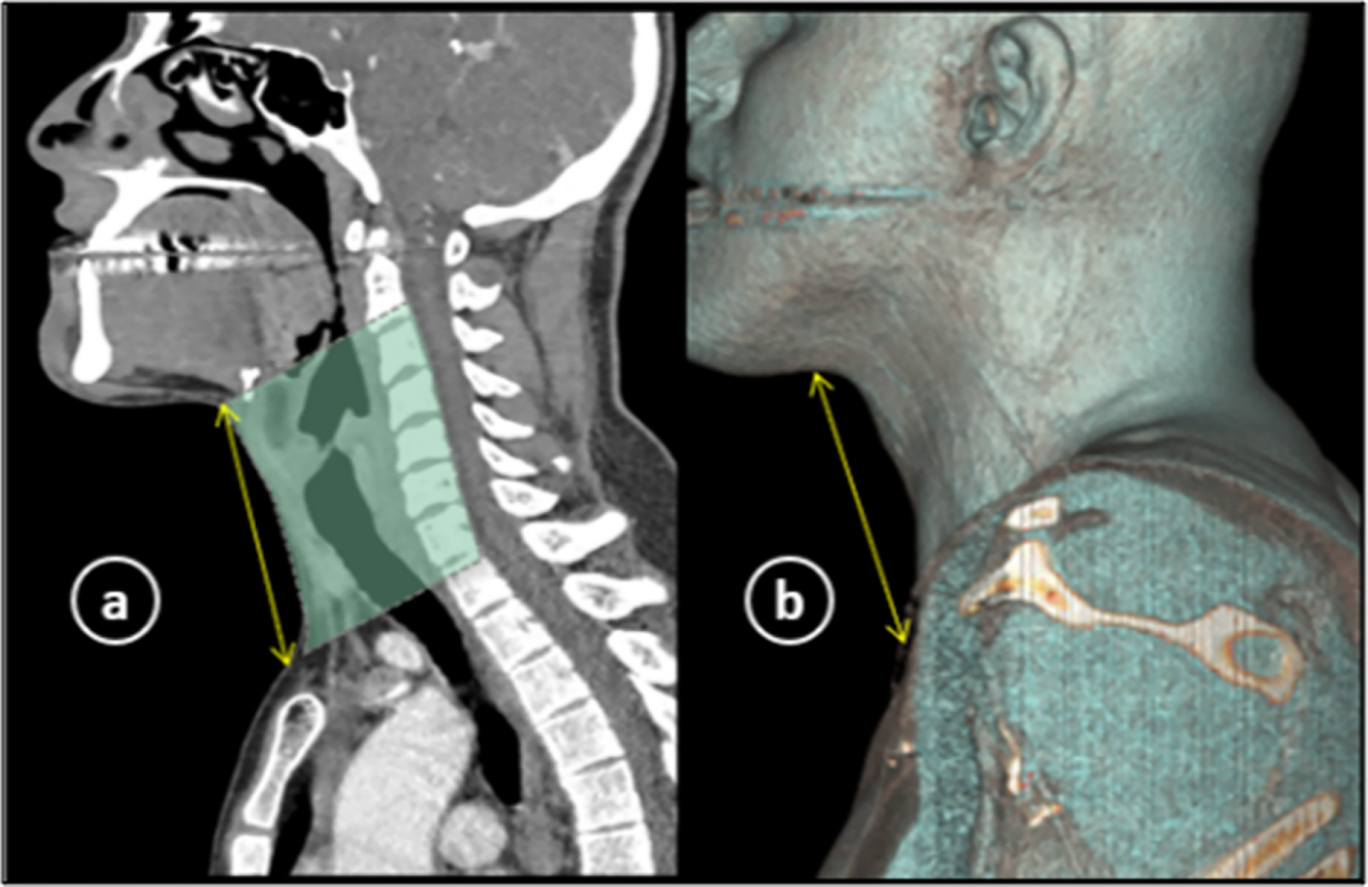
CT neck sagittal section (a) and its 3D volumetric reconstruction (b) showing how anterior neck length was measured (double arrow line).

Scans were examined on workstation by two radiologists, experienced in head and neck imaging, who were blinded for patient history. Initially sagittal plane was analyzed along with its 3D surface shaded reformat for anterior neck length (aNL) which was taken from lower border of chin till neck-chest junction at manubrium sterni (Fig 2).

All images were interpreted in soft tissue algorithm except for laryngeal cartilages evaluation. Glottic level was identified by placing localizer on cricoarytenoid joint. First, CT acquired data of NPVC-plane① was analyzed. Then, plane in sagittal section was adjusted parallel to vocal cord (VC) in order to achieve true axial plane, PVC- plane②. Following parameters in each plane were evaluated:

- Qualitative anatomical parameters: Vertebral level of axial plane, lumen shape, fat, split thyroid cartilage appearance and submental structures.
- Quantitative anatomical parameter: aNL, right and left VCw, ACom, AP, Tr and CSA.

Angle formed between plane① and ② was also noted for further analysis of relationship between measured parameters (Fig 1).

### Statistical analysis

Data analysis was performed using SPSS (IBM SPSS Statistics for Windows, Version 201.0 Armonk, NY: IBM Corp.). Descriptive data were expressed as mean ± standard error (SE) along with range (minimum and maximum). Qualitative data were presented as frequencies and percentages. Kolmogorov-Smirnov and Shapiro-Wilk tests were performed for normality. Different quantitative variables were compared by paired Student’s t- test and Chi-square test was used to compare categorical variables of study.

Spearman’s and Pearson’s correlations were computed where appropriate. Frequency of different glottic parameters including atypical shape, fat in glottis, open thyroid lamina, submental structure at glottic level and CT plane crossing more than one vertebral level were noted in each plane and they were dichotomized to see their impact on changing true anatomy of glottis by receiver operating characteristic (ROC) analysis and relative area under curve (AUC) were also recorded to see predictive value for them. *p* value of < 0.05 being considered statistically significant.

## Results

### Participants

**Fig 3:**
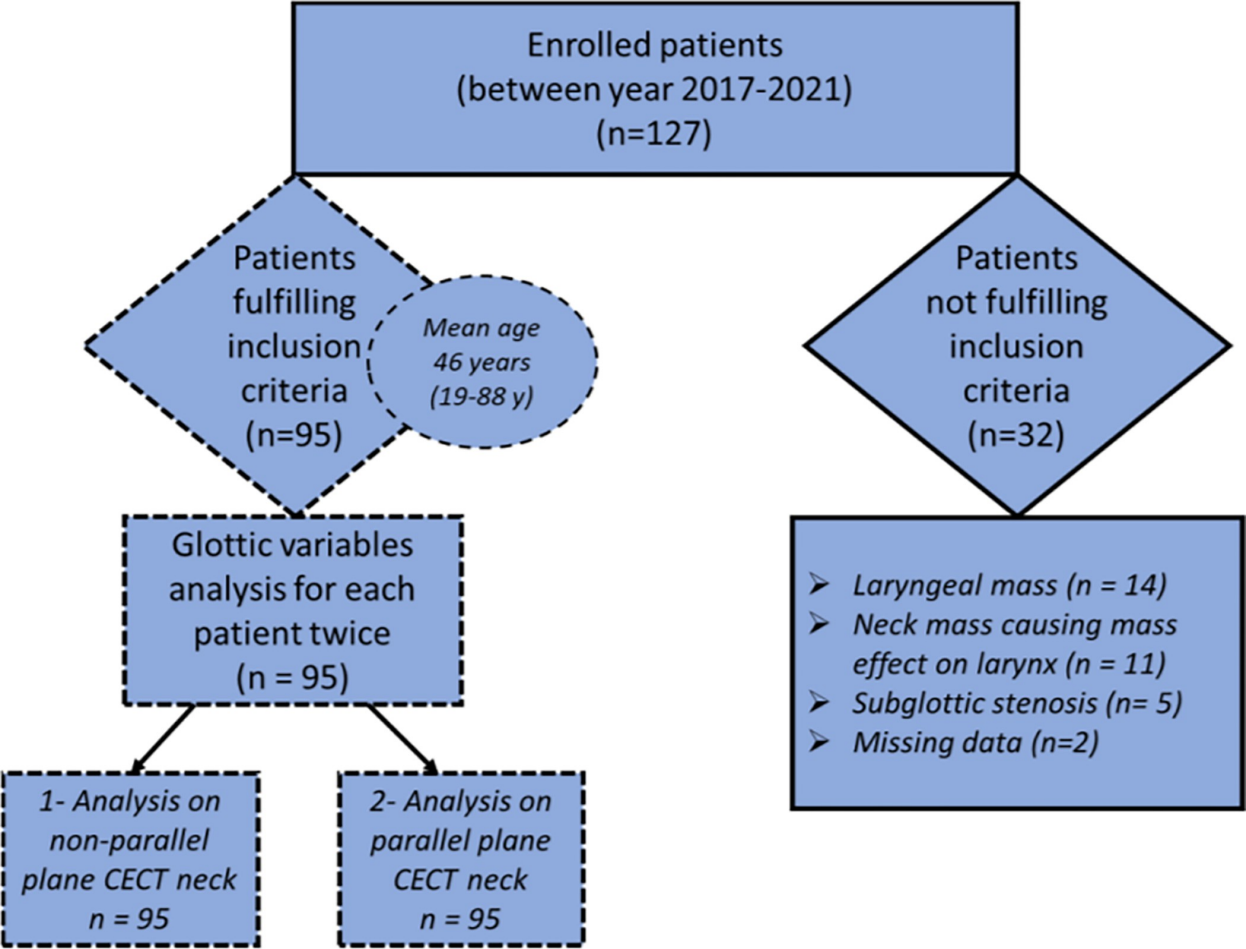
Flowchart summarising patient selection in this study. CECT = contrast enhanced CT scan.

A total of 127 patients’ files were scrutinized for eligibility criteria, out of which 32 were withdrawn from study leaving 95 patients (42 males, 53 females) to be further analysed. Excluded patients (32) were having laryngeal mass, neck mass with compression on larynx, subglottic stenosis and missing data (n = 14, 11, 5 and 2 respectively). Means (range, standard deviation) of aNL, age, height, weight and body mass index (BMI) of patients were 57.49 cm (10.5–100.8, ±19.1) 46.3 years (19–88, ±15.2), 162.6 cm (143–192, ±10.8), 79.5 kg (39–151± 21.5) and 29.9 kg/m^2^ (16.4–50.4,±7.1) respectively. Summary of patient selection is presented in flow chart (Fig 3).

### Comparative anatomy of qualitative glottic parameters

**Fig 4:**
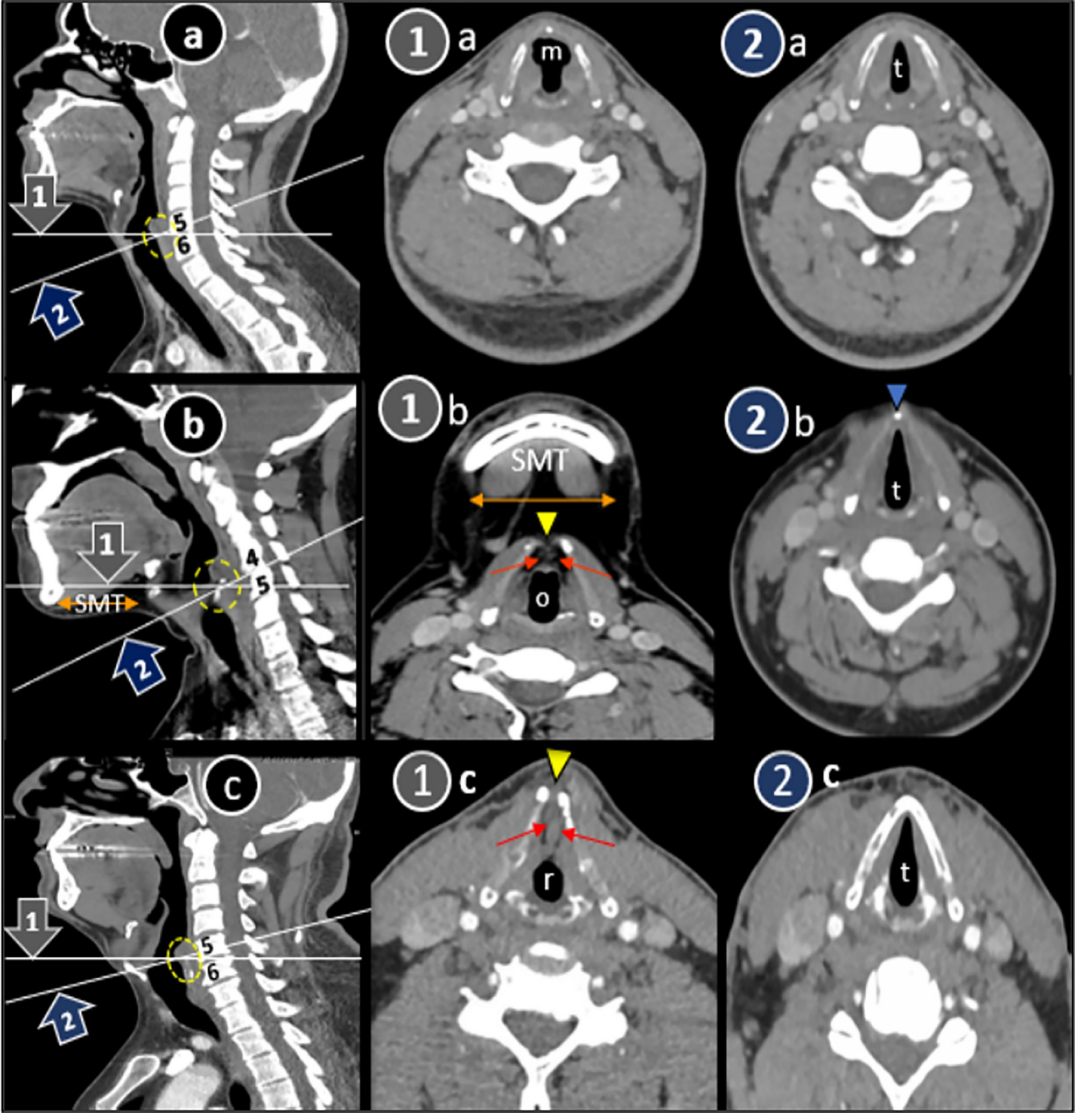
Sagittal post-contrast CT scan neck (a-c) showing two axial CT scan plane lines, non-parallel① and parallel to vocal cord ② intersecting at level of cricoarytenoid joint (dotted circle). Altered glottic parameters in plane① (1a-1c) are shown i.e., change in glottic lumen shape from tear (t) to mushroom (m), oval (o) and round (r), open and close thyroid laminae (yellow and blue arrowheads), inclusion of submental triangle (SMT, horizontal double head arrows) and adipose tissue (red arrows). Crossing of two axial planes (①,②) through cervical vertebrae (4,5,6) are also shown. Parallel plane② CT scans are also displayed for comparison (2a-2c).

**Fig 5:**
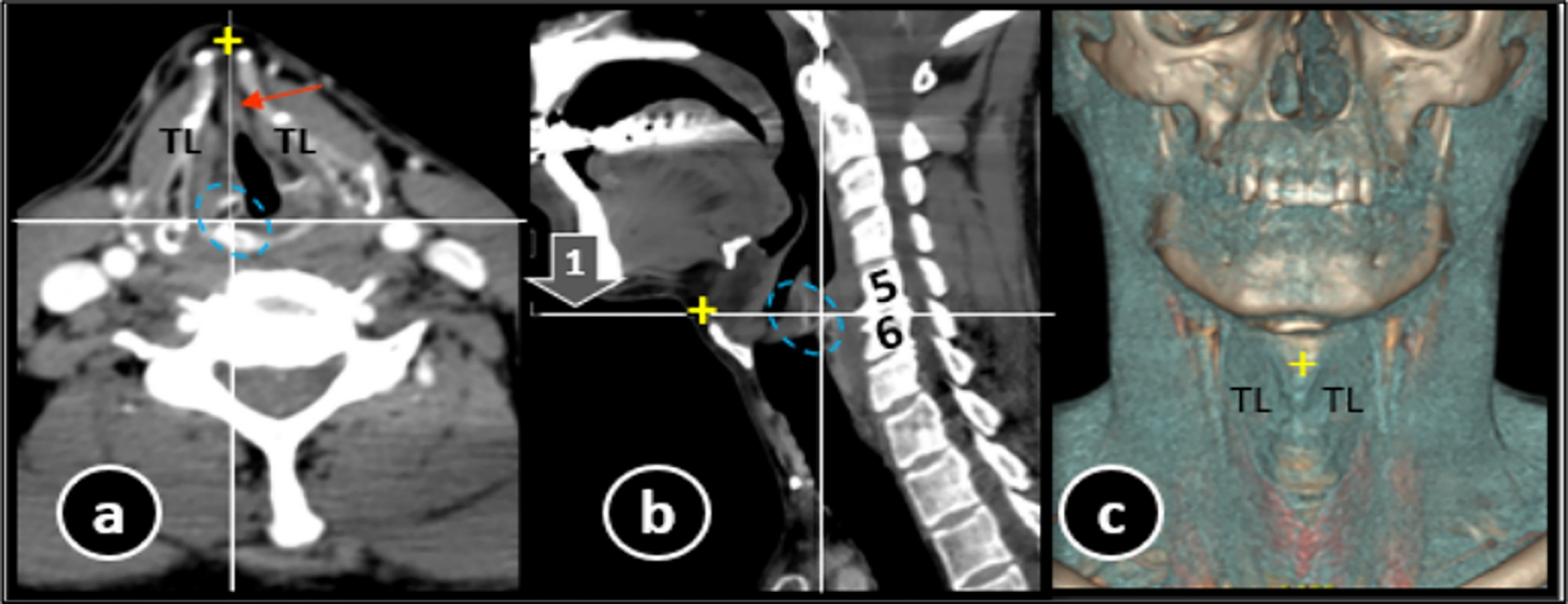
Axial (a) and sagittal (b) post-contrast CT neck showing glottis in non-parallel plane①. Split appearance of thyroid laminae (TL) shown by cross linking (+) this gap in a, b and 3D CT volumetric neck reconstruction. It is in actual a thyroid notch (c). Adipose tissue (red arrow), cricoarytenoid joint (dotted oval) and oblique crossing of plane① through vertebrae (5, 6) are also shown.

All showed statistically significant variation between their frequencies, suggesting that NPVC-plane① does not present true glottic appearance. Following are presented in Tables 2 and 3; Figs 4 and 5.

- True tear or wedge shaped *glottic lumen* was in only 25.3% of plane①. Other shapes were oval, mushroom and round.
- *Adipose tissue*, a part of false vocal cord, was present in 90.5% of true VC in plane①, but none in true plane.
- 70.5% of *thyroid laminae* had a central gap indicating plane① passing through thyroid notch (supraglottic level), but all planes② showed closed cartilage (glottic level).
- We do not expect *submental triangle structures* to be part of an axial section at true vocal cord level, but was noted in majority of plane①.
- Orientation of all planes② or VC were coinciding with cervical vertebrae or discs unlike plane① *crossing vertebrae obliquely*.

**Table 2:** Comparison of glottic appearance on CT scan in plane, non-parallel and parallel to vocal cord (n = 95).

No.	Parameters	Non-parallel plane CT plane① %(n)	Parallel plane CT plane② %(n)	*p* value
1	Shape	Tear 25.3(24), Oval 63.2(60), Mushroom 8.4(8), Round 3.2(3)	Tear 93.7(89) Oval 6.3(6)	0.0001
2	Presence of fat density	90.5(86)	0(0)	
3	Closed thyroid lamina	29.5(28)	100(95)	
4	Presence of submental structures	67.4(64)	7.4(7)	
5	Axial plane crossing >1 vertebral level	87.4(83)	0(0)	

**Table 3:** Area under curve (AUC) values of qualitative parameters of glottis.

Glottic parameters in NPVC plane①	Atypical glottic shape	Fat density in glottis	Open thyroid laminae	Presence of submental structures	Plane crossing >1 vertebral level
**AUC values**	0.652	0.740	0.686	0.781	0.736
**95% confidence limits**	0.530–0.775	0.528–0.953	0.567–0.805	0.685–0.877	0.585–0.888
***P* values**	0.018	0.018	0.004	0.000	0.008

Above glottic indicators were also analysed by ROC analysis which also demonstrated statistically significant number of CT scans exhibiting altered glottic anatomy in plane①. Out of these, presence of submental structures and manifestation of fat density in glottic sections were the foremost discriminators of erroneous representation of true glottis. Glottic shape and split appearance of thyroid laminae were next in consideration (Table 3).

### Comparative anatomy of quantitative glottic parameters

On application of paired t-test, numerical differences observed in each variable of vocal cord and glottic lumen showed high t-value and significant p value proposing unreliability of plane① for quantitative glottic analysis.

**Fig 6:**
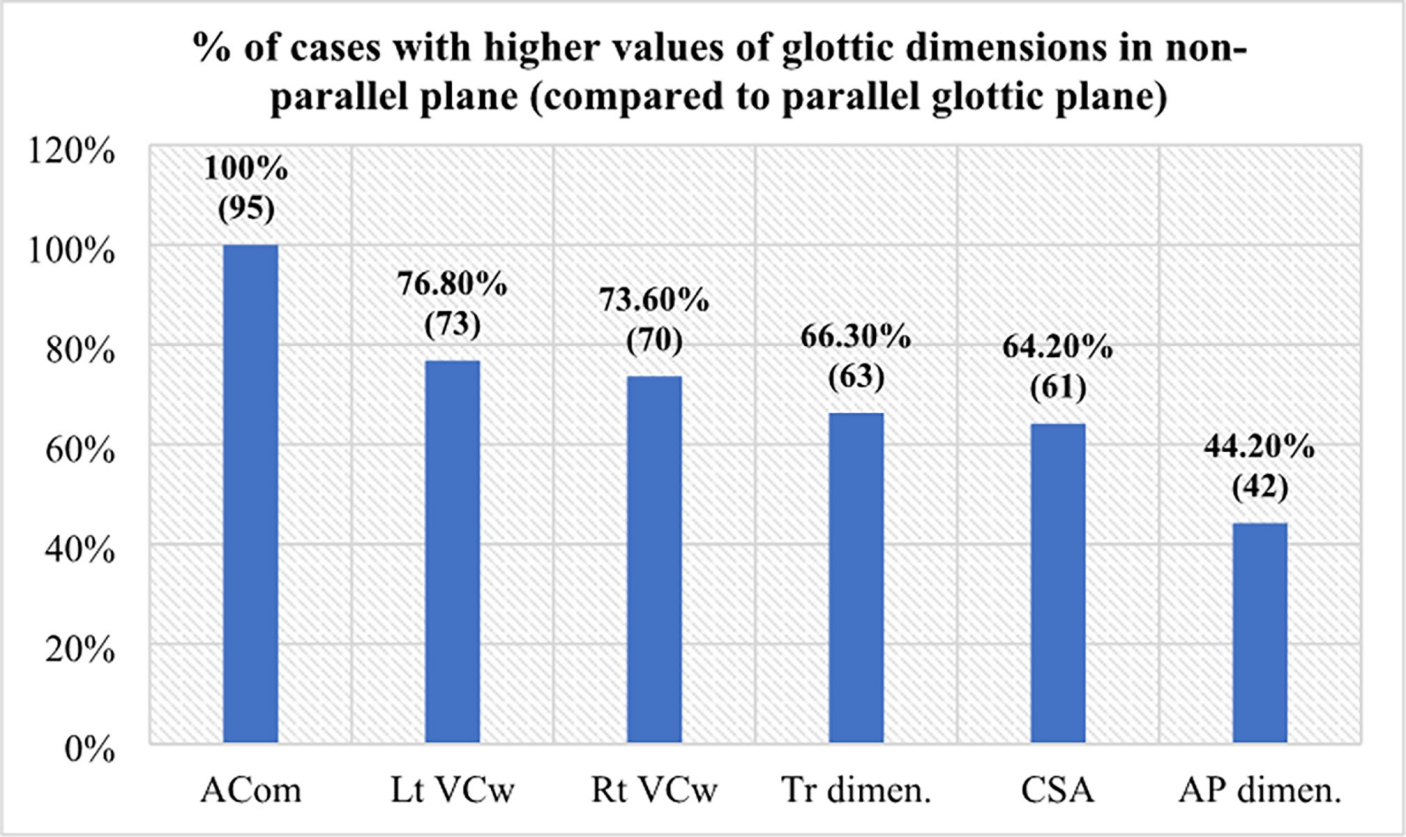
Bar chart illustrating percentages of cases in non-parallel plane① CT scan having higher values of glottic measurements. ACom = anterior commissure, VCw = vocal cord width, Tr = transverse width, AP = anteroposterior, dimen. = dimension, () = no.of patients.

Numerical differences in these parameters were calculated by subtracting plane② from plane① and exhibited both negative and positive values. Bilateral VCw showed mean variation of up to -0.7 mm. This variation was about -5.8 mm in ACom thickness with highest t-value. Although differences in AP and Tr dimensions observed between two planes were minor (1.27mm and -0.99 mm respectively), yet they were statistically significant. Out of glottic airway dimensions, CSA showed highest t-value and a mean difference of -15mm^2^. Majority of patients in plane① showed greater glottic dimensions than plane② (Fig 6) which is shown by their negative values. Exception was AP dimension because falsely thickened ACom in a normal glottis reduced AP size of glottic lumen because anterior lumen was being taken up by ACom (Tables 4 and 5).

**Table 4:** Comparison of glottic dimensions in plane non-parallel and parallel to vocal cord.

No.	Parameters n = 95	Non-parallel plane CT plane① Mean (SE)	Parallel plane CT plane② Mean (SE)	Mean diff (SE)	*t*-value	*P* value
	**Vocal cord (mm)**					
1.	Rt VC width	5.8 (0.16)	5.1 (0.13)	-0.75	-4.97	0.000
2.	Lt VC width	5.7 (0.15)	5.0 (0.10)	-0.72	-5.17	
3.	ACom	6.3 (0.44)	0.5 (0.05)	-5.8	-13.64	
	**Glottic lumen**					
1.	AP dimension (mm)	19.3 (0.40)	20.6 (0.43)	1.27	2.72	0.008
2.	T dimension (mm)	11.8 (0.34)	10.8 (0.23)	-0.99	-3.86	0.000
3.	CSA (mm^2^)	176.6 (6.14)	161.6 (5.61)	-15.0	-4.11	

Rt = right, Lt = left, VC = vocal cord, AC = anterior commissure, AP = anteroposterior, Tr = transverse, CSA = cross-sectional area, diff = difference, SE = standard error of mean

**Table 5:** Ranges of vocal cord and glottic dimensions with minimum (min) and maximum (max) differences among these parameters.

No.	Parameters N = 95	Non-parallel plane CT plane① (range)	Parallel plane CT plane② (range)	Min—max diff in parameters
	**Vocal cord (mm)**			
1.	Rt VC width	0.7–9.6	2.2–9.5	0–4.1
2.	Lt VC width	0.5–10.0	2.1–7.4	0–5.0
3.	ACom	0.2–23.4	0.1–2.1	0–23.3
	**Glottic lumen**			
1.	AP dimension (mm)	3.7–28.3	2.1–34.9	0–22.6
2.	T dimension (mm)	4.2–30.3	6.4–17.6	0–18.1
3.	CSA (mm^2^)	52.3–375.6	67.9–346.0	0.7–99.6

Rt = right, Lt = left, VC = vocal cord, ACom = anterior commissure, AP = anteroposterior, Tr = transverse, CSA = cross-sectional area, diff = difference, SE = standard error of mean

Ranges of glottic dimensions in each axial plane along with minimum and maximum differences in glottic measurements are provided in Table 5.

### Glottic parameters and body built versus obliquity of vocal planes

Spearman’s and Pearson’s correlations were determined to enquire how degree of deviation from plane② to① influenced glottic parameters.

Range of angle between plane① and ② was between 6.3° and 37° with mean of 23.2°± 6.6. This also indirectly discerned orientation of true vocal cords.

There was significant negative correlation (correlation coefficient (cc) = -0.27) (p = 0.008) between angle and vertebral level, implying that crossing planes of more oblique cords are at higher vertebral level. If plane ① angle was more deviated, it made more alteration in ACom thickness (cc = 0.2, p = 0.016), CSA (cc = 0.2, p = 0.049) and Tr diameter (cc = 0.3, p = 0.001). Additionally, change in one dimension influenced other measurements as well i.e., greater variation in ACom thickness and CSA values between these two planes showed more alterations in AP (cc = 0.3, p = 0.000) and Tr sizes of glottis (cc = 0.2, p = 0.022) respectively.

On correlation amongst dimensions of plane①, increased value of CSA significantly (p = 0.000) increased AP (cc = 0.5) and Tr (cc = 0.6) measurements but decreased VCw (cc = -0.3, p = 0.000). Thicker ACom reduced AP dimension, but insignificantly, (cc = -0.14, p = 0.155) which could be due to disproportionate variations between them in plane①. However, thick ACom showed more bilateral VCw (cc = 0.3, p = 0.000, 0.002), as ACom is product of bilateral cords.

**Fig 7:**
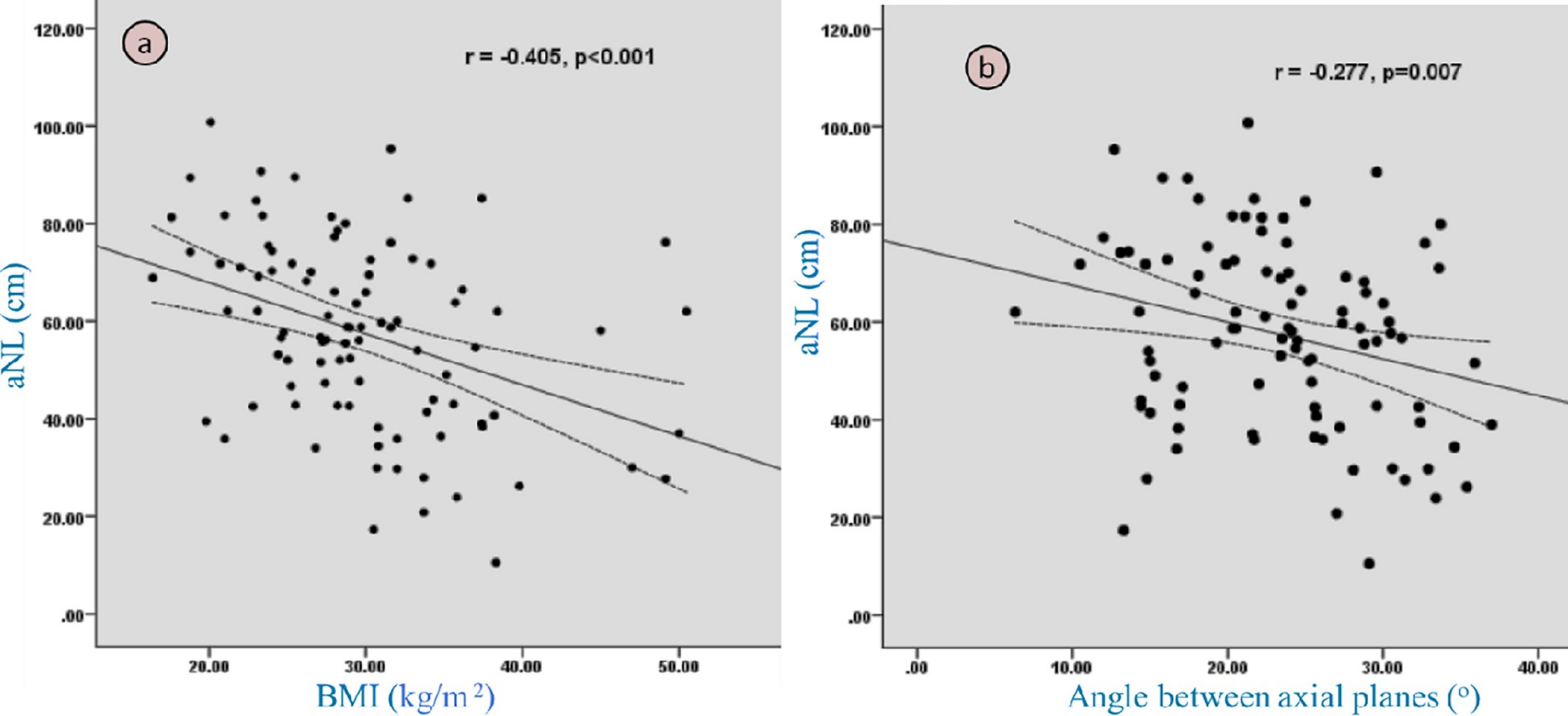
Scatter plot (a, b) showing negative correlation of anterior neck length (aNL) with Body mass index (BMI) and angle (°) between two CT planes (or cord angle).

Patients with high BMI were having short neck length (cc = -0.4, p = 0.000). Short necked patients were having more oblique or angulated cord (cc = -0.27, p = 0.007). BMI was found to have very weak insignificant relationship with VC angle directly (cc = 0.1, p = 0.26) (Fig 7).

## Discussion

Our study intends to determine effect of unformatted① CT on glottic morphological parameters by comparing it with standard features of glottis which are expected to be seen on true reformatted plane②. Non-adherence to definitive laryngeal CT protocol led to significant alterations in appearance and dimensions of glottis. Among all numerical differences, plane① impacted ACom and CSA the most. Vocal cord obliquity also imparted to extent of change in glottic variables and was related to neck length as well. To best of our literature search, no similar study has been published till date exploring an outcome of NPVC cross-sectional plane in terms of fallacious glottic anatomy and dimensions.

Post-endoscopic evaluation, CT scan is commonly an initial imaging encounter of various laryngeal disorders. MRI adds information (in <10%) if minor soft tissue and laryngeal cartilage details are required [6, 9]. For optimal diagnostic output, dedicated CT head and neck is recommended having display slice thickness of ≤ 3mm and axial plane parallel to vocal cord. Before advent of MPR, this angle is used to be selected preferably in preliminary lateral scout view by gantry angulation in plane of air in laryngeal ventricle, hyoid bone or cervical vertebrae [5, 11, 21]. Malpositioning of patient or scan angle may present images which obscure or simulate pathology and asymmetry. Here radiologist’s report acts as a key to ultimate patient management if they are able to differentiate between true and pseudoanatomy of glottis [6, 9, 11, 14, 23].

**Fig 8:**
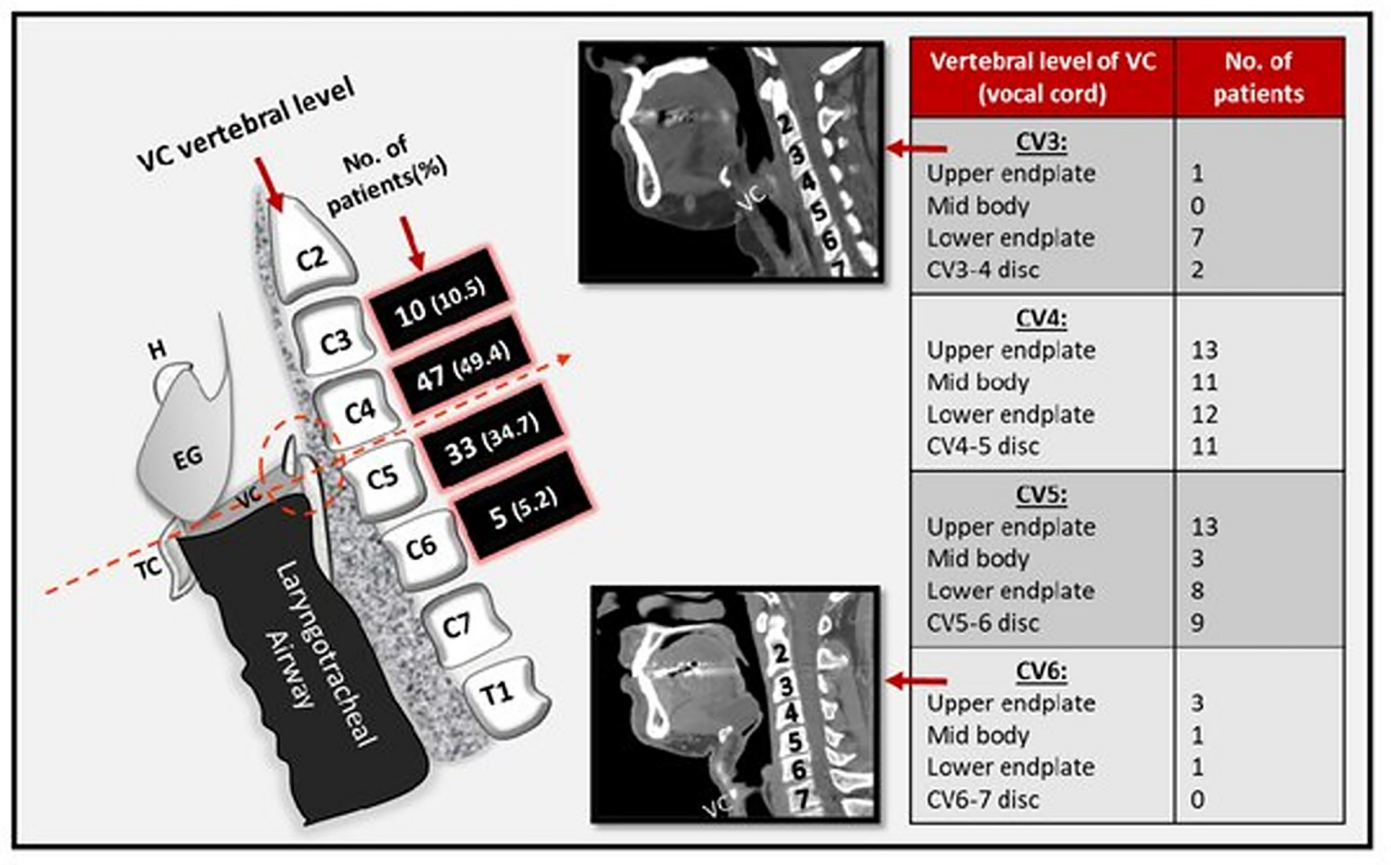
Diagrammatic representation of a larynx showing position of vocal cord (VC) in relation to cervical vertebrae (CV). Details about specific location of VC at each vertebral level is also tabulated.

Awareness about anatomical position of VC is an essential requirement of laryngeal framework surgeries, intubation decisions and their outcome [24–26]. Level of vocal cord is between cervical vertebra (CV) 4 to 6 [6]. We found VC lying between CV3 upper endplate and CV6 lower endplate with majority at CV4 level (Fig 8). A study on 20 patients found majority of VC opposite to CV5 and orthogonal to vertebral column [12]. One vertebral level variation from our study might be due to small sample size of this study. In keeping with literature, our VC orientation was also in harmony with vertebrae which is anteroinferior to posterosuperior [6]. Contrary to this, Cinar et al. found posterior vocal cord (2/3^rd^ males, 1/3^rd^ females) positioned lower than anterior commisure. Selection of cadaver larynges might be a limitation [25]. However, our 83 posterior plane① were lower than anterior and crossed obliquely through multiple vertebral levels between anterior part of CV3 inferior end plate and posterior part of CV7 upper endplate.

**Fig 9:**
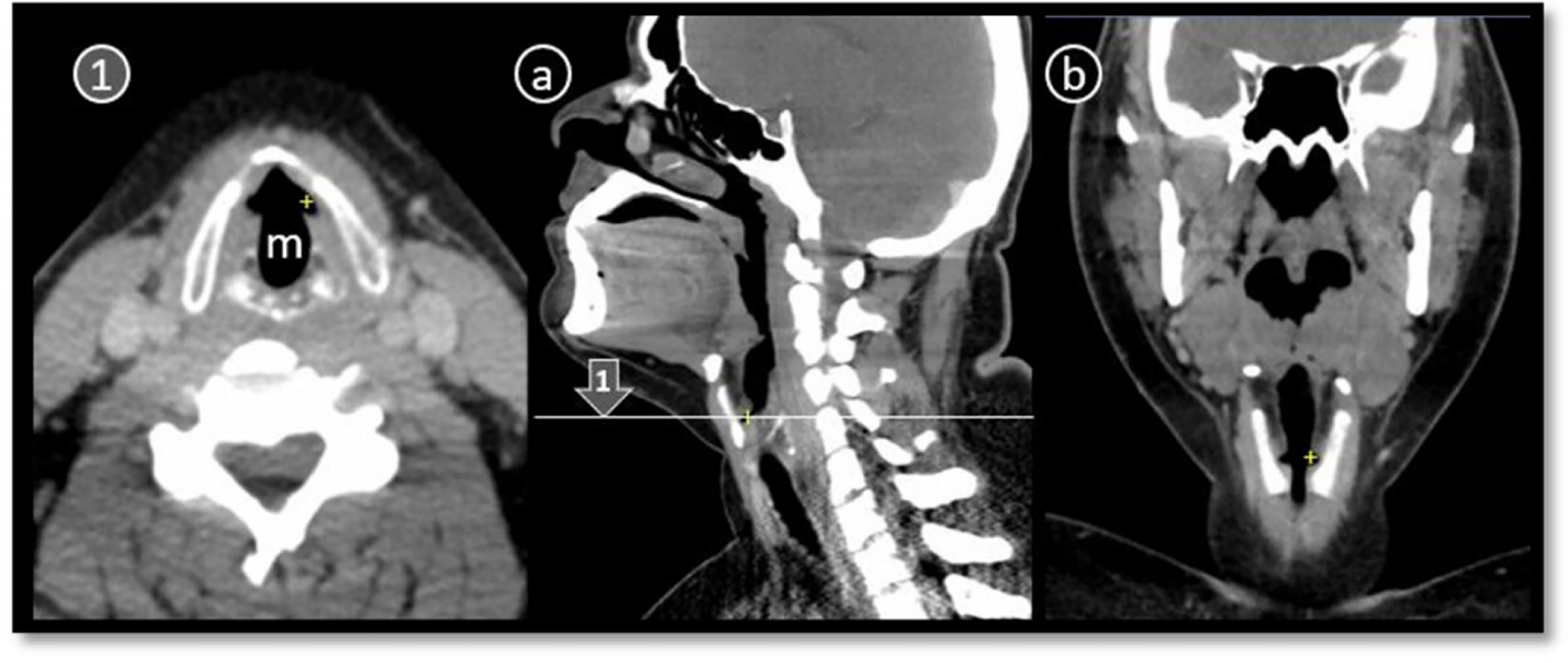
Post-contrast CT neck showing mushroom (m) shape of glottic lumen in non-parallel vocal cord (NPVC) plane① due to crossing of plane① (a) through laryngeal ventricle (+, a, b) rather than a true vocal cord. Left laryngeal ventricle is marked by + sign.

Glottis spans from upper margin of VC till distal arbitrary line at 10 mm [5, 27]. Radiological marking of this point is probably anterior lower triangular attachment margin of thyroarytenoid muscle. Axial view of CT scan equates with endoscopic picture at the level of cricoarytenoid joint and shows all glottic structures including true VC, ACom, posterior commisure, arytenoid cartilage (lower part), cricoid cartilage (upper part) and cricoarytenoid joint [6, 7]. Glottic lumen has a typical tear drop shape at this level due to triangular attachment of vocal cords [28]. Oval and round lumens do not justify its anatomy and mimic glottic stenosis. We noticed these shapes in 60 and 3 patients respectively. This was due to variable inclusion of anterior supraglottic tissues in NPVC plane causing increase in thickness of ACom, hence blunting anterior part of lumen shape. Glottis which showed maximum ACom thickness in our data (23.4mm) was round in shape. Mushroom lumen is a sign of unilateral vocal cord paralysis due to inclusion of ipsilateral distended ventricle sequela of medialization of posterior VC and contralateral anterior subglottic air [29, 30]. However, our 8 mushroom appearances were due to crossing of anterior NPVC plane through ventricular part of supraglottis bilaterally rather VC (Fig 9).

Fat is a content of false VC, pre-epiglottic and paraglottic spaces and indicates supraglottis [27]. CT scan can differentiate between adipose and thyroarytenoid (TA) muscle of glottis both subjectively (hypodense) and objectively (negative Hounsfield Unit value) [5, 28]. In a healthy larynx, presence of TA muscle soft tissue density in a fat at the level of glottis can be misinterpreted as a pathology [9]. Fat seen in our >90% of glottides were located either only anteriorly in expected ACom area or extended anterolaterally in VC region. This variation was dependent on obliquity of non-parallel plane causing variable inclusion of supraglottic fat in glottis(Fig 4①B and 4①C).

VC is attached at midline junction of upper two fifth and lower three fifth thyroid laminae thus designating superior thyroid notch as a level of supraglottis [25]. This notch has a split thyroid laminae appearance in axial view which can be misinterpreted as a cartilage invasion in pathological larynx [8], (Fig 5) In our study, 86 and 67 glottis showed supraglottic fat and superior thyroid notches respectively because of higher anterior orientation of plane①.

To our knowledge, there is no description in literature about presence or absence of submental structures at axial section level of true glottis. Excess adipose accumulation in submental region is regarded as a known phenotype of obesity leading to low positioned inferior chin border [31]. High frequency of submental structures or fat (67.4%, p = 0.0001) visibility in plane① might be due to major distribution of high BMI patients in our data set (76.8%) rather than effect of plane. However, result is statistically significant. Hence it can be implied that submental structures are not part of axial glottic plane and their presence should alert a radiologist to reassess CT neck reformatting (Fig 4①B).

Influence of angle between two planes on above five discussed physical glottic parameters was also inspected. We first counted number of qualitative changes in parameters out of five followed by recording angle ranges which come with each number of change from 1–5. If angle was between 14.9° to 37° there was 100% change in variables, while none if it was <14°. From this conclusion can be drawn that more inclined plane ① is more prone to show false glottic anatomy and potentially pathology (Table 6).

**Table 6:** Tabulated representation to show influence of angle between PVC and NPVC planes (deviation angle from true plane) on 5 parameters of glottic anatomy.

No. of parameters changed (out of 5)	No. of patients (%)	Angle of deviation between planes
**5**	32 (33.6)	14.9–35.9°
**4**	36(37.8)	12.7–34.6°
**3**	16(16.8)	14.4–33.4°
**2**	6(6.3)	10.5–25°
**1**	3(3.1)	12–17.4°
**0**	2(2.1)	6.3° & 14.3°

PVC = parallel vocal cord, NPV = non-parallel vocal cord, 5 parameters: Shape, fat density, thyroid notch, submental structures, vertebral level of vocal cord

**Fig 10:**
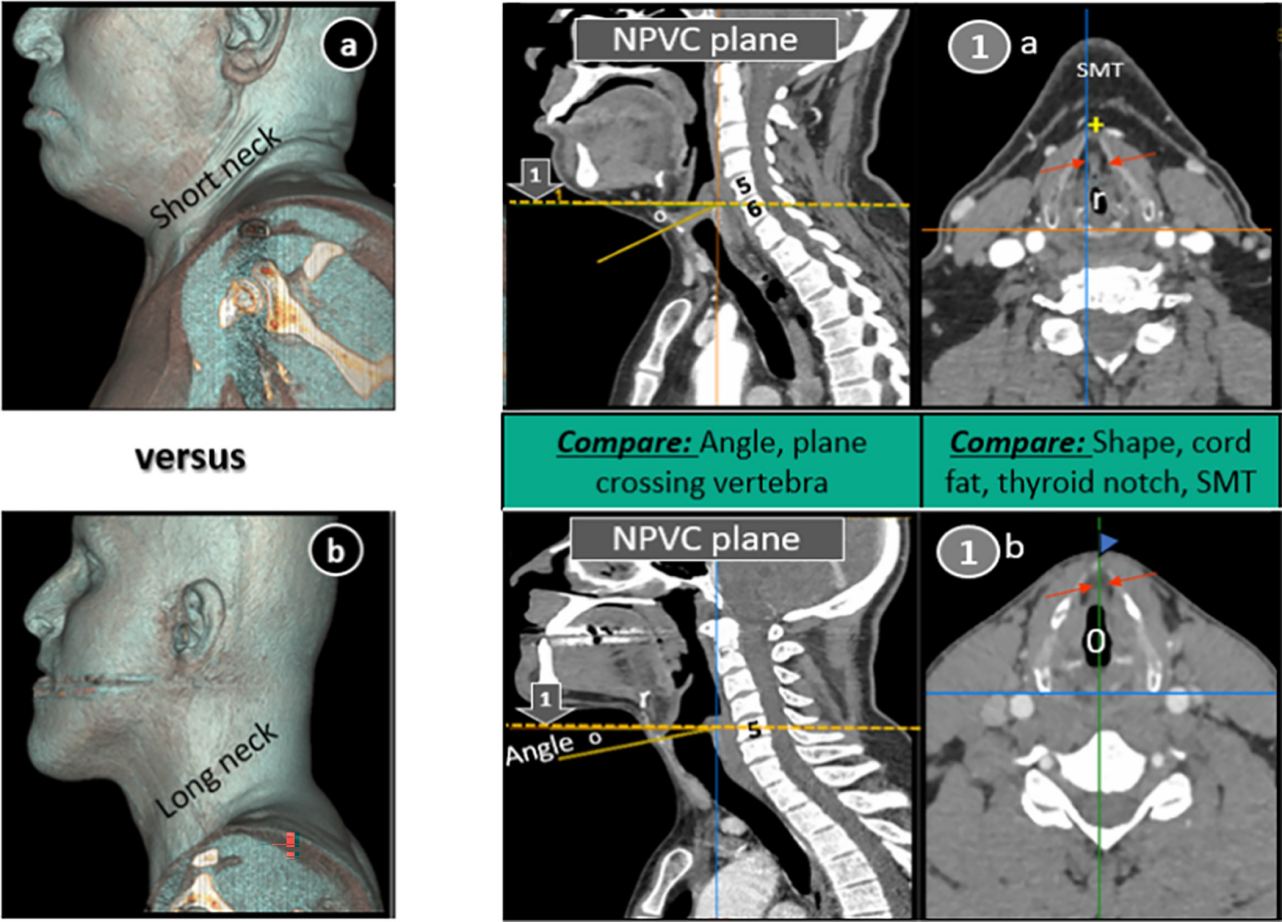
3D CT volumetric neck reconstruction of short versus long neck (a, b) along with comparison of their necks on sagittal and axial CT scans. Obliquity of non-parallel vocal cord (NPVC) plane (o) and variation of plane crossing vertebrae are shown in sagittal sections indicating more in short neck. Visualisation of structures which are not part of glottis are seen more in short neck (1a) than long neck (1b). Different shapes (round, r/oval, 0) affect quantitative parameters as well.

Here relationship drawn between BMI, neck length and cord angle is worth mentioning. We found significant negative correlation between BMI versus neck length and neck length versus angle, demonstrating that unformatted images of obese patients with short necks were more prone to show up false glottic anatomy due to higher angulation between two planes or steeper VC in them (Fig 10). Thus, radiologist and laryngologist should opt meticulous approach to assess CT necks of these patients as short necks without CT reformation not only compromise radiological anatomy but laryngoscopic evaluation of airway as well [32].

Precise glottic morphometry is vital to improve diagnostic and therapeutic procedures outcome in a field of laryngoscopy [33]. Glottic lumen is bounded laterally by vocal folds, anteriorly by junctional zone of vocal cords i.e., anterior commissure and posteriorly by posterior commisure between arytenoid cartilages [27, 34, 35]. Measured dimensions of its lumen can vary either due to pathology or various physiological factors including phonation, breath holding, swallowing, demographic characteristics or CT technique [9, 10, 36, 37]. We have not encountered a single study mentioning all glottic parameters in non-cadaveric sample of CT scan. Our empirical data of vocal cord showed that mean right and left VCw in PVC plane are 5.1mm and 5.0 mm, while 5.8mm and 5.7 mm in NPVC plane respectively. Jotz et al. calculated cord width in corps stating it to be 6.07 mm in males and 5.03 in females [38]. Conversely, other studies reported 4.2 mm, 8.22 mm in males and 3.1 mm, 7.43 mm in females with high *p* values in later [33, 39]. Hu et al. found VC width as 6.23 mm in males and 4.90 mm in females on ultrasound [40]. Despite dynamicity in sonographic measurements, they can be compared with axial CT measurements. Our VCw values taken in untrue plane① were closer to above-mentioned values in literature which is explainable because of selection of non-representative laryngeal samples in them i.e., cadavers and dynamic ultrasound. Cadaveric organ has a major constraint of tissue contraction due to chemical preservation. Ultrasound captures VCw alterations during different phases of breathing. CT NPVC plane cuts thyroarytenoid VC muscle obliquely rather parallel resulting in higher dimensions than a true plane. With modern concept of focal vocal cord radiotherapy by using CT volume in early glottic carcinomas, these limitations and our true measurements might be helpful [19].

ACom imaging demands cautious assessment because it is regarded as a major indicator for treatment decisions and prognostic outcome of laryngeal masses. It is considered abnormal if anteroposterior dimension is ≥ 2.1 mm. Studies proposed its normal thickness ranging from immeasurable (<1 mm) to 2.2 mm. This is comparable to our study which is between 0.1–2.1 mm [34, 35]. Reporting on unformatted images can show up its maximum thickness up to 23.4 mm with a mean value of 6.3 mm, in an otherwise normal looking larynx. Mean value of ACom in plane② was 0.5 mm (Tables 4 and 5). 97.9% of ACom was >2.1mm thick in NPVC plane, with or without fat density in it. In our data, ACom thickness had an inverse effect on AP dimension in plane ① as AP measurement of anterior lumen is occupied by ACom. This relationship has potential to falsely represent glottic lumen size.

Calculations of airway lumen has been debated over the years because of its importance in airway management, surgical planning and endotracheal tube selection. Mean AP, Tr and CSA measurements of glottic lumen in PVC plane were 20.6 mm, 10.8 mm and 161.6 mm^2^ compared to 19.3 mm, 11.8 mm and 176.6 mm^2^ in NPVC plane respectively. All above measurements were statistically significant. Aljathlany et al. investigated upper airway dimensions on CT scan and reported these glottic dimensions as 22.8 mm, 11.3 mm and 170 mm^2^ respectively [16]. All luminal dimensions are close to our NPVC plane except AP dimension questioning an accuracy of plane selection. They also derived comparison of computation method for CSA analysis from the same data set and preferred software manual method, as used in our study [36]. Our observation of negative relationship between CSA and VCw is self-explanatory as thick VC reduces lumen size. However, large CSA of glottic lumen has bigger AP and Tr dimensions.

Main limitation of our study is constraint of variability of laryngeal morphometry with neck position, phase of respiration and phonation. Due to retrospective analysis, we could not get uniform slice thickness of CT reformation, but all were ≤3mm. This study being peculiar, we could not find any reference values in literature to compare our results in NPVC plane. Moreover, we studied glottis at one particular level ignoring other laryngeal levels, but this could be extended for future. Although sample size was appropriate and representative of population in terms of statistically significant results, nonetheless larger cohorts are proposed to reinforce an impact of inaccurate CT neck techniques. Checklist to recognise false glottic CT anatomy due to plane ① (Table 7) and highlights about possible imaging reporting pitfalls under the light of literature is unique to our study which can be considered in future for analysis of its application in laryngeal pathologies especially tumors (Table 8).

**Table 7:** Checklist of qualitative features (at glottic level), which if present identifies pseudoglottic level due to non-parallel vocal cord plane.

Checklist to identify inaccurate level of true vocal cord on CT neck
✓ Atypical shaped glottic lumen ✓ Presence of fat density ✓ Open thyroid laminae ✓ Thick anterior commisure with fat ✓ Axial plane passing obliquely through cervical vertebral or disc plane ✓ ± Appearance of submental structures

**Table 8:** Possible pitfalls which can be encountered when interpreting images of larynx in non-parallel CT larynx plane.

Potential pitfalls while reporting non-parallel vocal cord plane CT larynx
**Qualitative assessment** • Normal glottis having variable shapes can be misinterpreted as vocal cord paralysis or glottic stenosis • If supraglottis adipose (seen in glottis) is occupied by mass, it will be considered as glottic in origin • Normal thyroarytenoid muscle in supraglottis fat can be misinterpreted as a mass • Falsely thickened anterior commissure (ACom) having pathology can be taken as ACom in origin rather anterior supraglottis • Open thyroid lamina can be misreported as thyroid erosion especially in tumors
**Quantitative assessment** • Vocal cord and glottic lumen dimensions if imprecise can lead to various miscalculations in laryngeal interventions • Wrong size of laryngeal lumen can be a cause of incorrect choice of endotracheal tube • Non-representative glottic plane in laryngeal masses can lead to inaccurate tumor dimensions and its mislocalisation, so is tumor staging.

## Conclusion

Unformatted CT neck exhibits fallacious anatomical structures and inaccurate dimensions of a larynx at level of a glottis, hence does not represent true depiction of glottis. This finding also supports recommended CT neck protocol and intimating radiologists to verify laryngeal imaging reformation before interpretation of scans. This may further be helpful for laryngologists to choose a pertinent management decision. Points which need to remembered are listed in Table 9.

**Table 9:** Points need to be remembered by radiologist and laryngeal surgeons while interpreting laryngeal CT.

Points to remember
• CT of larynx parallel to vocal cord plane is basic requirement for optimal radiology reporting. • Before reporting, CT images should be scrutinised for accurate technique through identification of false glottic structures. • Non-parallel CT scan changes dimensions of airway lumen and can represent false anatomy and hence dissatisfactory management decisions. • Laryngeal surgeons also need to collaborate and review images before taking any morbid management decision.

## Supporting information

S1 Data (XLSX)
