# Correction: Novel and pragmatic exploration of variation in glottic parameters in non-parallel versus parallel vocal cord CT planes with potential reporting pitfalls

**DOI:** 10.1371/journal.pone.0296606

**Published:** 2024-01-02

**Authors:** Adeena Khan, Waleed M. S. Fawzy, Syed S. Habib, Mamoona Sultan

There are errors in the Funding section. The correct Funding statement is: The authors extend their appreciation for the Deputyship for Research and Innovation “Ministry of Education” Saudi Arabia for funding this research (IFKSUOR3-210-2).
